# Targeting cardiomyocyte proliferation as a key approach of promoting heart repair after injury

**DOI:** 10.1186/s43556-021-00047-y

**Published:** 2021-11-05

**Authors:** Shuainan Li, Wenya Ma, Benzhi Cai

**Affiliations:** 1 Department of Pharmacy at The Second Affiliated Hospital, and Department of Pharmacology at College of Pharmacy (The Key Laboratory of Cardiovascular Medicine Research, Ministry of Education), Harbin Medical University, Harbin, 150086 China; 2 Institute of Clinical Pharmacy, the Heilongjiang Key Laboratory of Drug Research, Harbin Medical University, Harbin, 150086 China; 3 Research Unit of Noninfectious Chronic Diseases in Frigid Zone, Chinese Academy of Medical Sciences, Harbin, 150086 China

**Keywords:** Cardiomyocyte proliferation, Heart regeneration, Myocardial infarction, Cardiovascular disease, MicroRNAs, Cardiac repair

## Abstract

Cardiovascular diseases such as myocardial infarction (MI) is a major contributor to human mortality and morbidity. The mammalian adult heart almost loses its plasticity to appreciably regenerate new cardiomyocytes after injuries, such as MI and heart failure. The neonatal heart exhibits robust proliferative capacity when exposed to varying forms of myocardial damage. The ability of the neonatal heart to repair the injury and prevent pathological left ventricular remodeling leads to preserved or improved cardiac function. Therefore, promoting cardiomyocyte proliferation after injuries to reinitiate the process of cardiomyocyte regeneration, and suppress heart failure and other serious cardiovascular problems have become the primary goal of many researchers. Here, we review recent studies in this field and summarize the factors that act upon the proliferation of cardiomyocytes and cardiac repair after injury and discuss the new possibilities for potential clinical treatment strategies for cardiovascular diseases.

## Introduction

Cardiovascular disease (CVD) is the leading cause of patients' death in the world, and Asia accounts for nearly half of CVD cases [1–4]. The majority of CVD happens to the elderly, presenting as myocardial ischemia [5], which usually develops into myocardial infarction (MI) and heart failure with irreversible pathological changes [6, 7]. Although surgical treatments can alleviate severe CVD, it has non-negligible limitations, such as organ insufficiency and postoperative complications [4], and also fail to replenish cardiac myocytes lost in the injured heart. Therefore, the researchers are committed to finding ways to repair the damaged heart by increasing the number of endogenous cardiomyocytes [8, 9]. Studies have shown that cardiomyocytes can recover their proliferative capacity through the regulation of certain factors [10–14]. Thus, promoting adult cardiomyocyte proliferation is considered a new hope and an encouraging therapeutic strategy for treating myocardial ischemic disease.

Gene expression is the process by which genetic information is transferred from DNA to proteins through transcription and translation. The precise control of gene expression in all cells, i.e. the final formation of proteins, is necessary for the functioning of the organism. Therefore, changes in the expression of proteins (including transcription factors) will inevitably have an impact on endogenous cardiac regeneration. In recent years, several transcription factors, extracellular matrix proteins, and soluble factors have been continuously identified to be involved in cardiomyocyte proliferation and cardiac repair [15–19]. In addition to protein-coding genes, non-coding RNAs such as microRNAs are also involved in regulating cardiac regeneration [20, 21]. Furthermore, signaling pathways such as NRG1/ErbB, Notch, Hippo/YAP, Wnt/β-catenin have been reported to be even more essential in cardiac repair after injury [22–25]. These results have undeniable reference significance to support therapeutic strategy of heart repair by targeting cardiomyocyte proliferation.

Here, we present an overview divided into several sections: transcription factors, extracellular matrix, signaling pathways, soluble factors, and microRNAs. In this article, we sort out the latest research advances in each field in cardiac regenerative repair and discuss the controversial contents, which have potential implications for the study of regenerative drugs.

## Evidence of cardiomyocyte proliferation and challenges in detecting cardiomyocyte proliferation

The first organ formed during embryogenesis is the heart [26]. Although the development of the heart is a complex process, cardiomyocyte proliferation is the main source of cardiac growth during embryonic development. Cardiomyocytes proliferate along the heart tube wall and atrial septum, with the highest proliferation rate occurring on the outer surface of the heart, the dense zone [27]. The epicardium is a thin layer of cells that envelops the heart and provides a source of mitogenic signals that stimulate cardiomyocyte proliferation within the dense zone [28]. For a long time, it has been generally believed that mature cardiomyocytes of mammals no longer have the ability to proliferate, and the main form of growth is an increase in cell size and muscle fiber density, rather than an increase in the number of cardiomyocytes [29].

**Fig. 1:**
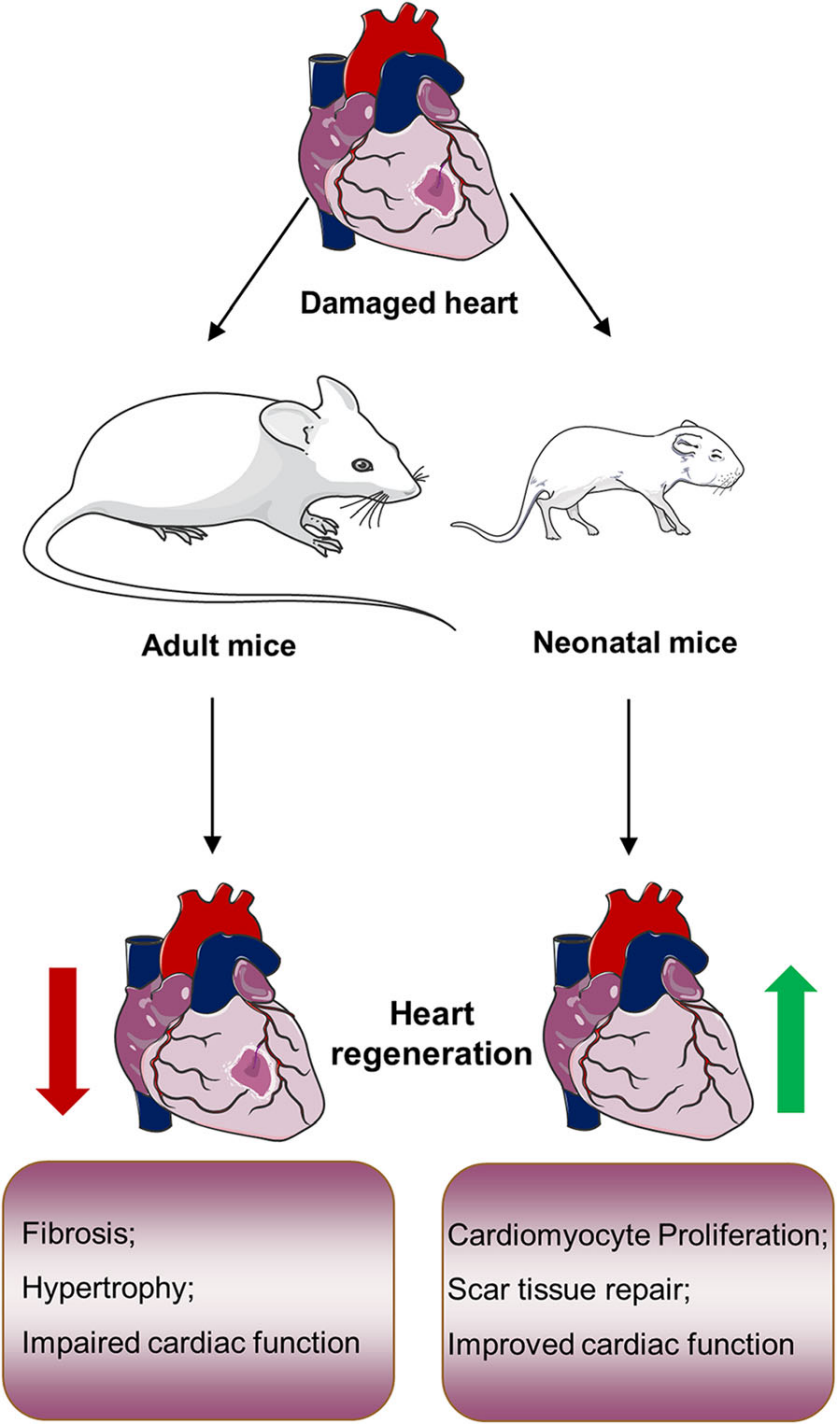
Overview of heart regeneration in mice inside and outside the regeneration window. After cardiac damage, neonatal mice exhibit promotion of cardiomyocyte proliferation, scar tissue repair, and improvement in cardiac function. Conversely, adult mice exhibit myocardial fibrosis, hypertrophy, and impairment of cardiac function

Initially, in zebrafish and newt, the injured heart could achieve efficient heart regeneration, as evidenced by the re-formation of functional cardiomyocytes at the site of injury and the elimination of myocardial scars after injury [30–33]. Interestingly, Porrello et al. reported that newborn mice have minimal scars at the heart injury site 21 days after AR (apical resection) and MI surgery, but this ability only exists in the heart of mice within 7 days of birth [29]. It suggests, during heart development, mammals have an excellent ability of regeneration, and the damaged tissue can repair itself through the renewal of myocardial cells, and this ability is rapidly lost as the heart tissue matures after 7 days of birth [29]. This period of self-healing is called the regeneration window (Fig. 1). Moreover, it is exciting that Bergmann et al. found that new cardiomyocytes can also be produced in the human adult heart, although the annual turnover rate of cardiomyocytes is low and decreases with age from 1% (25 years old) to 0.45% (75 years old) [34]. Not only that, early studies have shown that there is a renewal of cardiomyocytes in the marginal and distal regions of human myocardial infarction, even though the mitotic index is low [35]. Therefore, the origin of these cardiomyocytes involved in renewal during heart regeneration became the next major question for the researchers. Although many assumptions have been made before, most of the results ultimately point to the conclusion that newly formed cardiomyocytes during zebrafish and mammalian heart regeneration originate from the dedifferentiation and proliferation of pre-existing cardiomyocytes rather than from stem or progenitor cells [36–39]. These studies break the presumption that adult mammalian cardiomyocytes are unable to proliferate.

Currently, the application of proliferation markers and cardiomyocyte-specific antibodies is the main way to visualize and quantify cardiomyocyte proliferation. Ki67, EdU/BrdU, pH3, and Aurora B are the more widely used cell cycle proliferation markers. Ki67 protein is present in all phases except G0 [40]; EdU/BrdU, as a thymidine nucleoside analogue, an S-phase marker incorporated into replicated DNA molecules by replacing thymine [41, 42]; phosphorylation of histone 3 (pH3) is closely associated with chromosome cohesion in early mitosis and therefore serves as an M-phase marker [43]. Nevertheless, there are non-negligible problems in detecting cardiomyocyte proliferation. One of them is that the enhanced expression of some markers is not sufficient for the activation of the complete cell cycle. It has been showed that only about one-third of cells entering the S phase during the regeneration process of adult Newt cardiomyocytes entered mitosis and cytokinesis, suggesting that some markers do not mark the complete cell division [44]. With the application of Aurora B kinase, a key marker of the cytoplasmic division involved in chromatin segregation, this problem seems to have been solved [45, 46]. Another problem is that the phenomenon of binucleated or multinucleated adult cardiomyocytes makes it more difficult for us to detect true cardiomyocyte proliferation. Surprisingly, Hesse et al. showed that midbody localization and the daughter nucleus distance could distinguish true cardiomyocyte division and binuclear events [47]. Despite this, the precise detection method is still poorly applied.

Although a small number of cardiomyocytes proliferate after adult mammalian heart injury, it is clear that these cardiomyocytes cannot achieve heart self-repair, so finding the triggers that can amplify the effect of proliferation are key to cardiac regeneration studies. In this paper, we summarize the triggers for cardiac regeneration in recent years.

## Transcription factors

Transcription factors can control chromatin and transcription by recognizing specific DNA sequences. It acts a pivotal part in controlling the biological process of cells and regulates the process of many diseases. Yet, the regulatory role of transcriptional networks in cell cycle arrest of cardiomyocytes after birth remains incompletely elucidated. In this part, we summarize the transcription factors which are demonstrated to affect cardiomyocyte duplication.

The first transcription factor identified to be involved in cardiac regeneration was Meis1, and Meis1 knockout prolonged the reproductive period of neonatal mouse hearts and promoted adult mouse cardiomyocytes to re-enter the cell cycle [48, 49]. Furthermore, overexpression of Meis1 inhibited cardiomyocyte proliferation, impeded myocardial repair, and increased scar area in neonatal mice after MI [48]. Nguyen et al. discovered that Hoxb13 was a cofactor of Meis1, as cardiomyocyte-specific deletion of Hoxb13 could produce similar effects as Meis1. Besides, Meis1-Hoxb13 double knockout significantly promoted cardiomyocyte proliferation and improved cardiac function after MI in adult mice, suggesting that the two factors can act synergistically to influence cell cycle progression and cardiac regeneration [50]. It is worth mentioning that Mesi1 also plays an important role in angiogenesis, indicating that it can have multiple effects on heart regeneration [51].

GATA4 is another essential transcription factor for cardiac development. In zebrafish hearts, injury triggered GATA4 expression within a week, and cells that previously expressed GATA4 marked most of the myocardium during the subsequent regeneration process [37]. Malek et al. demonstrated that the expression of Ccna2, Ccne1, CDK4, Cenpa, E2F1, and other cell cycle-related genes were down-regulated in the myocardium of GATA4-KO mice [52]. Cardiac-specific GATA4 knockout mice showed enhanced myocardial scarring after injury and the reduced proliferation of cardiomyocytes than their control, indicating impaired regeneration. In adenovirus-mediated overexpression of GATA4 mice, cardiac function was significantly improved after injury at p7 [52]. Additionally, GATA4 could promote cardiomyocyte replication and coronary angiogenesis by regulating the paracrine factor FGF16 [53]. Moreover, GATA4 also played a pivotal role in NF-κB signal-mediated heart regeneration [54].

Tbx20 is also involved in cardiomyocyte proliferation and cardiac homeostasis in adult hearts [55–57]. Studies have shown that Tbx20 expression is increased at the edge of injury in the adult zebrafish heart and that overexpression of Tbx20 induces dedifferentiation and proliferation of cardiomyocytes and stimulates the expression of cardiac fetal gene programs [58]. Tbx20 induced cardiomyocyte proliferation through multiple pathways, for instance activating BMP2/pSmad, PI3K/AKT/β-catenin signaling pathways, and the Hippo/YAP pathway. Besides, Tbx20 could also inhibit the expression of p21, Meis1, and Btg2 which are negative regulators of proliferation.

As mentioned above, cardiomyocytes exit the cell cycle permanently soon after birth. E2F transcription factor plays a crucial part in regulating cell cycle progression and growth [59]. Mehregan et al. found that the expression level of E2F-6 (E2F transcription factor 6) mRNA remained unchanged, while the protein level gradually decreased throughout the development until adulthood, indicating that E2F-6 plays a role in cardiomyocytes through post-transcriptional regulation. At the same time, the reduced expression of E2F-6 protein was closely related to the retreat of the cell cycle in cardiomyocytes, and the loss of E2F-6 expression resulted in a dramatic reduction in the survival rate, which suggests that E2F-6 is indispensable to maintain normal cardiomyocyte growth [60]. Coincidentally, Judd et al. found that E2F-2 (E2F transcription factor 2) could induce adult mouse cardiomyocytes to re-enter the cell cycle [61]. Chen et al. showed E2F-1 upregulated the expression of ECRAR, which is a promoter of cardiomyocyte proliferation and heart regeneration, and ECRAR stimulated activation of E2F-1 by facilitating phosphorylation of ERK1/2 [62]. The positive feedback pathway suggests a key role of E2F-1 in cardiac regeneration. In recent years, it has been found that the E2F/Rb transcriptional network is associated with mononucleation or binucleation in cardiomyocytes, and the induction of binucleation suppresses the expression of E2F target genes, suggesting an important association between E2F and nucleation [63]. This provides a basis for subsequent cardiac regeneration studies.

Casz1, a Zinc finger transcription factor, is a novel 1p36 coronary heart disease gene [64]. The level of Casz1 mRNA was easily measured during heart development [65]. The loss of Casz1 in the heart induced abnormal cardiac gene expression, cardiomyocyte proliferation reduction, ventricular septal defects (VSD) and cardiac morphological defects, and begot to heart failure and fetal death [66]. Dorr found that Casz1 exerted a vital part in the progression of the cell cycle in mammalian primary and secondary cardiac domains. The loss of Casz1 resulted in the prolongation or stagnation of the S phase, the decline of DNA synthesis and mitosis, and the significant downregulation in the cardiomyocyte population [67]. It demonstrates that Casz1 is required for the renewal of myocytes in the primary and secondary cardiac regions, and normal cardiac development and function in mammals. In addition, the interaction between Casz1 and TBX20 is necessary to maintain cardiac homeostasis and survival, and the lack of interaction between the two can lead to dilated cardiomyopathy [68].

## Extracellular matrix

Extracellular matrix (ECM), as the indispensable scaffold around cells, is a key part of all cells and tissues. Most importantly, it is closely related to the connection and regulation of various forms of cellular physiological activity. Studies have shown significant changes in ECM-related gene expression in regenerative newt hearts, implying an important role of ECM in cardiomyocyte proliferation [69]. However, the role of ECM in mammalian cardiac regeneration and repair remains largely unknown.

It has been shown that alteration of the extracellular matrix in the first week after birth can influence the growth and differentiation of mouse cardiomyocytes [70]. Agrin, as a proteoglycan, is an ingredient of the postnatal extracellular matrix. Recombinant Agrin could attenuate maturation and induce proliferation of myocardial cells originated from mouse and human iPSC. *In vivo*, Agrin could promote the reduction of infarct area and cardiac repair after myocardial damage in adult mice. Mechanistically, Agrin-induced cardiac regeneration was associated with activation of downstream YAP and ERK signaling pathways [71]. Baehr et al. further validated the effects of Agrin in an ischemia-reperfusion model in pigs and showed that local (antegrade) delivery of Agrin significantly reduced infarct size and improved inflammatory response and cardiac function after MI [72].

In a report, follistatin-like 1 (FSTL1) in the epicardium induced cell cycle re-entry of cardiomyocyte and protected the heart function from myocardial ischemia injury. Moreover, human FSTL1 (hFSTL1) synthesized in bacteria can induce cell proliferation, but not in mammals, demonstrating that post-translational modifications of hFSTL1 protein (e.g. glycosylation) affect its effect on regeneration. The function of specific N-glycosylation sites of hFSTL1 in cardiac regeneration was studied using the modified mRNA technique, and researchers detected that the mutation of hFSTL1 (N180Q) could increase cardiomyocyte proliferation in mice. Collectively, administration of N180Q hFSTL1 modRNA to the myocardium could promote the proliferation of profuse cardiomyocytes and protected the heart from myocardial infarction [73]. AltekoEster also discovered that the delivery of FSTL1 to the impaired heart activated the proliferation of myocardial cells and the recovery of cardiac function [74]. Wei and colleagues confirmed that FSTL1 delivered via bioengineered collagen patches, as an epicardial secretory factor, had protective effects on the injured heart [75].

Fibulin family, of which seven genotypes are currently known, is widely present in basement membranes as a class of secreted ECM glycoproteins. Researchers found that Fibulin-1 inhibited the proliferation of trabecular cardiomyocytes by suppressing the activation of ErbB2 and ERK1/2, thereby maintaining normal ventricular development [76]. In addition, Tsuda et al. revealed that Fibulin-2 deficiency reduced mortality in MI mice, mainly by decreasing TGF-β signaling to attenuate inflammatory cell infiltration and the frequency of cardiac rupture, thereby reducing the occurrence of left ventricular dysfunction [77].

Cysteine-rich 61 (CCN1) is a member of the CCN family. Feng et al. showed the important role of CCN1 in cardiac regenerative repair; specifically, CCN1 induced proliferation of neonatal mouse cardiomyocytes after AR by promoting fibroblast senescence. Furthermore, CCN1 promoted fibroblast senescence after MI in adult mice thereby reducing fibrosis and improving post-injury cardiac function [12].

The expression of periostin, high content in the ECM of rat myocardium, decreases gradually from embryonic to adult periods [78]. Kühn et al. first found that *in vitro* periostin induced the re-entry of differentiated cardiomyocytes into the cell cycle, as evidenced by increased expression of cardiomyocyte proliferation markers, and *in vivo* periostin improved cardiac function after MI and promoted cardiac repair. In addition, the regenerative effect was due to the direct action of periostin on cardiomyocytes rather than other cells [79]. However, Lorts et al. came to the contradictory conclusion that the increase or decrease of periostin content in the heart after an injury did not affect DNA synthesis, mitosis, and cytoplasmic division, so periostin could not induce cardiac repair. They speculated that the cardioprotective effects might be due to its influence on collagen fiber formation and ventricular remodeling [80]. Subsequently, Polizzotti et al. reported that administration of recombinant periostin peptide promoted the proliferation of cardiomyocytes and cardiac repair after MI in pigs [81]. In a recent study, Chen et al. came to similar conclusions that knockdown of periostin inhibited cardiomyocyte proliferation and revascularization and promoted myocardial fibrosis after MI in neonatal mice. They showed that this regenerative effect was closely linked to the PI3K/GSK3β/cyclin D1 signaling pathway [82].

Besides, decellularized ECM treatment has beneficial effects on repair after cardiac injury. The use of fetal ECM culture *in vitro* significantly promoted the proliferation of rat ventricular myocytes [83]. *In vivo*, injection of decellularized zebrafish cardiac ECM and neonatal mouse cardiac ECM facilitated cardiomyocyte proliferation and cardiac repair after MI, accompanied by activation of ErbB2 receptors [84, 85]. The mechanical stiffness of the myocardium changes post-acute myocardial infarction (AMI). After an anterior apical infarction, the myocardium first becomes stiffer followed by greater compliance [86]. Furthermore, the researchers found that the mechanical stiffness of the local microenvironment was strongly correlated with the regenerative capacity of the heart; specifically, a reduction in ECM stiffness restored the regenerative capacity of mice after AR [87]. Recent studies have shown that decellularized fetal ECM combined with the reduction of microenvironment stiffness could significantly promote cardiomyocyte proliferation and improve cardiac function after injury, and mechanical unloading could significantly enhance the regeneration of decellularized fetal ECM by enhancing nuclear YAP signal [88].

In summary, we found that both direct action on cardiomyocytes and indirect beneficial effects on the heart indicate the importance of ECM in this field. Moreover, ECM patch studies have made great progress and several clinical trials are underway, illustrating the broad prospects of ECM in cardiac regenerative repair [18, 89].

## Signaling pathways

**Fig. 2:**
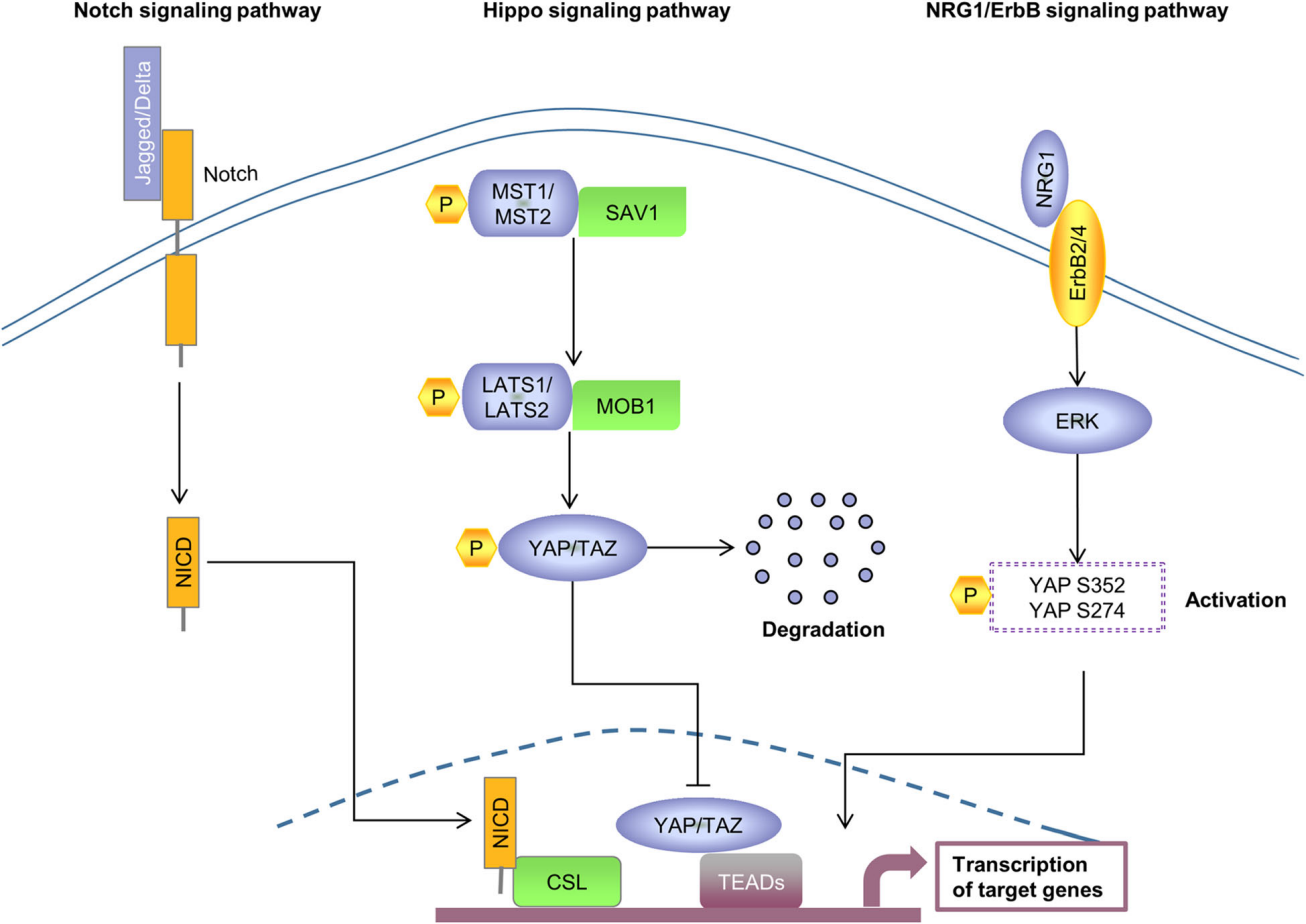
A sketch of the working model and interactions of the Hippo, Notch, and NRG1/ErbB signaling pathways. Specifically, Notch signaling pathway activation by Jagged/Delta binding to Notch followed by two protein hydrolysis releases NICD into the nucleus, and then NICD binds to CSL for targeting gene expression. However, Hippo signaling is activated by cascade phosphorylation of core kinases (MST1/MST2, SAV1, LATS1/LATS2, MOB1), which subsequently phosphorylate YAP and TAZ so that they cannot enter the nucleus to bind to TEAD family members. In addition, there is crosstalk between ErbB signaling and Hippo signaling, as ErbB signaling activates ERK and thus phosphorylates the S352 and S274 sites of YAP to promote transcription of target genes; incidentally, this works by acting on the non-classical Hippo/YAP pathway

**Fig. 3:**
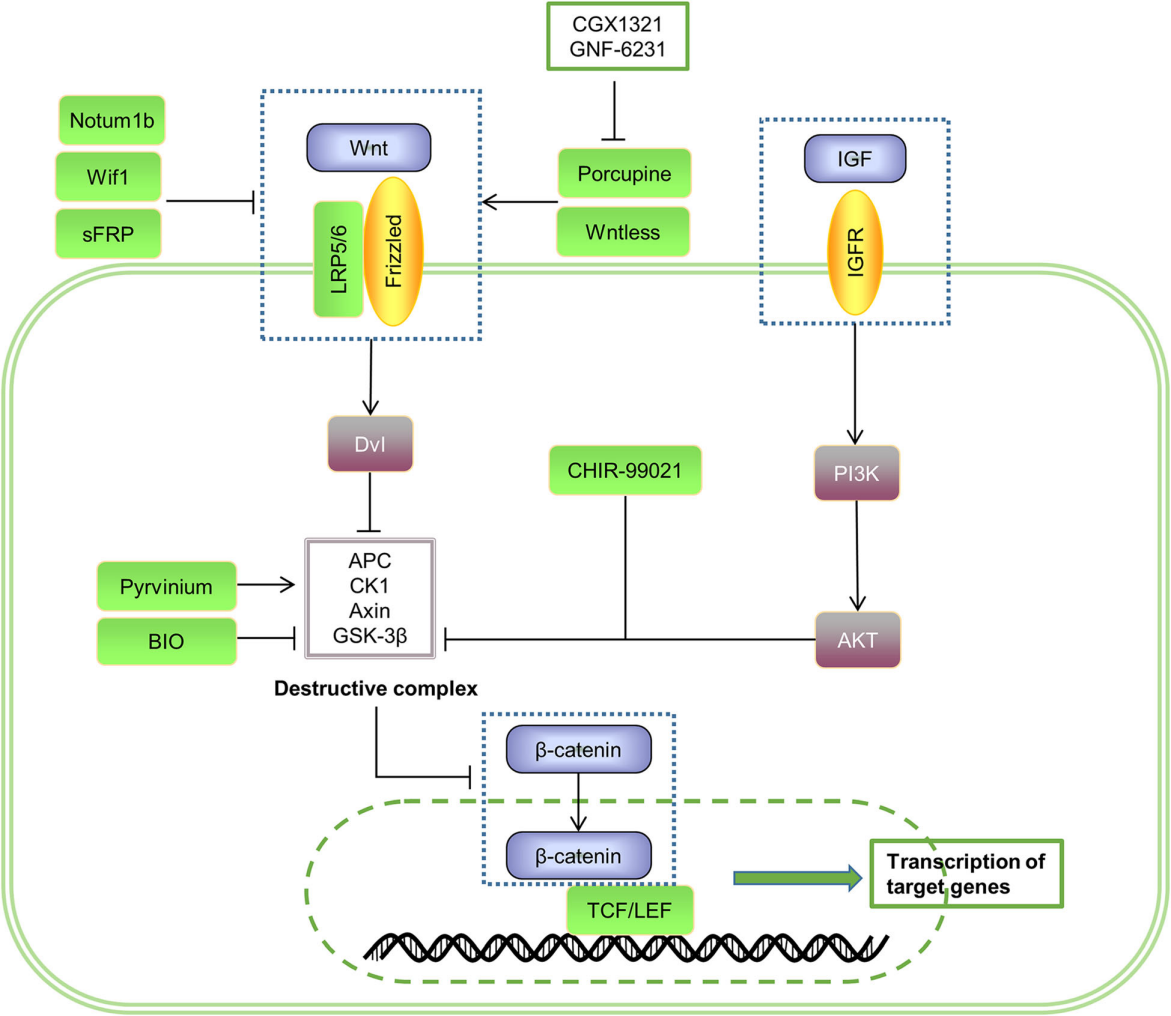
The classical Wnt/β-catenin signaling pathway in cardiac regeneration. After binding of Wnt ligands to their membrane receptors Frizzled and LRP5/6, Dvl inhibits the degradation of the disruption complex and stabilizes β-catenin in the cytoplasm. The increased β-catenin in the nucleus binds to TCF/LEF and regulates the transcription of target genes. In addition, the activation of IGF signaling during this process promotes the stabilization of β-catenin by affecting PI3K/AKT to inhibit the expression of GSK-3β

In recent years, besides transcription factors and ECM proteins, signaling pathways are also engaged in the process of cell cycle delay and cardiac regeneration restart. The relevant signaling pathways identified so far include NRG1/ErbB, Notch, Hippo/YAP, Wnt/β-catenin, etc (summarized in Figs. 2 and 3).

### NRG1/ErbB signaling

It was recently found that the upregulation of neuromodulin-1 (NRG1) facilitated cardiomyocyte proliferation and cardiac repair through its receptor ErbB2 in injured zebrafish hearts [22, 90]. Furthermore, cardiac-specific ErbB2 knockout affected cardiomyocyte proliferation at embryonic/neonatal stages; impaired NRG1/ErbB2 signaling pathways limited proliferation and regeneration after birth and affected heart integrity and cardiac function [91]. Therefore, the NRG1/ErbB2 pathway is imperative for cardiomyocyte proliferation and sufficient to activate the heart regenerative window [92]. Honkoop et al. indicated that NRG1/ErbB2 induced cardiomyocyte proliferation after injury in mice and zebrafish by mediating metabolic reprogramming, suggesting a possible impact of glycolysis in cardiac regeneration [93]. Interestingly, one study reported crosstalk between NRG1/ErbB and Hippo/YAP signaling. ErbB2 activated a non-classical YAP pathway to regulate cardiac regeneration after HF; the specific mechanism was to promote ERK activation and epithelial-mesenchymal transition-like mechanical changes to phosphorylate two key sites (S352 and S274) of YAP [94]. Similarly, Bersell et al. found that NRG1/ErbB4 signaling stimulated cardiomyocyte proliferation and regeneration of cardiac repair after injury in adult mice [95]. This signaling pathway is also widely used in the treatment of other heart diseases. NRG1 treatment significantly restored cardiac function in heart failure animals and patients [96]. Also, substances that activate the NRG1/ErbB2 pathway can also exert cardioprotective effects. For example, astragalus polysaccharide exerted protective effects against diabetic cardiomyopathy by activating NRG1/ErbB and AKT/PI3K; exercise increased the level of NRG1 and thus protected cardiac function in rats after infarction [22, 97].

### Notch signaling

Notch signaling is evolutionarily conserved and closely associated with the development of many organs, especially the heart. This is evidenced by the detection of mutations in Notch genes (Notch1/2/3) in many cardiovascular diseases [98, 99]. The Notch signaling pathway is mainly mediated by the binding of Notch ligands to Notch receptors, and then the notch intracellular domain (NICD) is released into the nucleus through two protein hydrolysis to regulate downstream genes. Notch ligands include Jagged 1/2 and Delta 1/3/4; Notch receptors include Notch1/2/3/4 [100].

Notch signaling was first identified to be involved in regulating cardiac regeneration in the zebrafish heart [101]. Notch signaling was activated in the endocardium and epicardium after injury and both inhibition and overactivation of Notch receptors inhibited cardiomyocyte proliferation and impaired regeneration in zebrafish [102]. In addition, Zhao et al. found that inhibition of endocardial Notch signaling after zebrafish injury led to scar formation and suppressed cardiomyocyte proliferation, whereas activation of Notch signaling promoted cardiac regeneration by producing Wif1 and Notum1b in the endocardium to inhibit the Wnt signaling pathway [103]. Similar to zebrafish, activation of Notch signaling was observed in MI-induced myocardial injury models in mice and rats, suggesting a protective effect of Notch signaling against cardiac injury [104, 105]. *In vitro*, activation of Notch 1 signaling promoted proliferation and inhibited apoptosis in rat cardiac-derived H9C2 cells by regulating Bcl-2/Bax as well as caspase-9/-3 [106]. *In vivo*, activation of Notch signaling, either by overexpression of NICD1 or Jagged1 or by other pathways, promoted the proliferation of neonatal murine cardiomyocytes [99, 107–109]. However, whether it affects adult cardiomyocytes proliferation after MI remains controversial. Kratsios et al. observed that after Notch signal activation, Ki67-positive cardiomyocytes increased after myocardial infarction in adult mice, but pH3 did not change [99]. Furthermore, Felician et al. concluded that Notch signaling activation was ineffective in adult murine cardiomyocytes after MI due to a closed chromatin state of the Notch-responsive promoter, which manifested as repressive chromatin enrichment and high levels of CpG methylation. In addition, the researchers demonstrated that activation of the Notch pathway could not promote post-infarction cardiac repair by promoting adult cardiomyocyte proliferation, but could exert beneficial effects on the heart through other cells, providing another potential idea of Notch regulation of adult mammalian heart regeneration [108]

### Hippo/YAP signaling

The Hippo signaling pathway is an evolutionarily conserved key pathway that regulates the size of tissues and organs [110–114]. The major components of the Hippo signaling pathway are first reported in Drosophila and subsequently identified in mammals, and the two are highly homologous. Specifically, MST1/MST2, SAV1 (also known as Salv), LATS1/LATS2, MOB1A/MOB1B, YAP/TAZ, and TEADs in mammals are homologous to Hippo, Salvador, Warts, Mats, Yorkie, and Scalloped in Drosophila, respectively [110, 115–123]. Briefly, the main pathways of hippo signaling are as follows: upon hippo signaling activation, the MTS1/MTS2 and SAV1 complexes promote the activation of phosphorylation and interaction between LATS1/LATS2 and MOB1, which mediates YAP/TAZ phosphorylation for cytoplasmic degradation. In contrast, when hippo signaling is inactivated, the transcriptional co-activator YAP/TAZ enters the nucleus and synergistically regulates the expression of target genes with transcription factors such as TEADs, thus acting to control organ size and cell proliferation [114, 123–127].

Hippo signaling is critical in the normal development of the heart. It was found that inactivation of the Hippo signaling pathway by cardiac-specific knockout (CKO) Salv in the embryo resulted in increased cardiomyocyte proliferation, and most Salv CKO mice died after birth and exhibited excessive cardiac enlargement [127]. In contrast, inactivation of YAP at the embryonic stage suppressed cardiomyocyte proliferation and showed severe cardiac hypoplasia and lethality [126, 128]. It suggests that the Hippo/YAP signaling pathway is necessary to maintain cardiomyocyte proliferation and normal heart size development. In neonatal mouse hearts, YAP1 CKO resulted in decreased cardiac function and premature death; YAP CKO impaired the inherent regenerative capacity of neonatal mice post-MI, manifested as severe myocardial fibrosis [129, 130]. However, in mouse hearts that exited the regenerative window of P8, Salv CKO significantly promoted cardiomyocyte proliferation, reduction of the scar area, and recovery of cardiac function after MI/AR; in adult hearts, the researchers obtained similar findings as in P8 hearts [131–133]. In the non-stressed adult mouse heart, both Lats1/2 and Salv CKO and cardiac-specific activation of YAP promoted the proliferation of adult cardiomyocytes that had withdrawn from the cell cycle without affecting cardiac function [131, 134]. Under stress (MI), nuclear YAP1 was found in the infarct border zone rather than the remote zone [130]. It implies that YAP plays an important role in adult heart repair. As expected, after MI in adult mice, knockdown of YAP1 inhibited cardiomyocyte proliferation, promoted apoptosis, and exacerbated myocardial fibrosis resulting in severely impaired cardiac function; *in vitro*, overexpression of YAP1 induced cardiomyocyte proliferation and reversed H_2_O_2_-induced cell death [130]. Similarly, *in vivo*, both activation of YAP and deletion of Salv drove the expression of cell cycle proliferation-related genes and improved cardiac function and survival after MI/iHF (ischemic HF) in adult mice, specifically manifested as the increase of cardiomyocyte proliferation, the recovery of pumping function, the weakening of fibrosis, and the enhancement of contractility [129, 134, 135]. To better understand the regulatory role of Hippo pathway components, we have detailed the mouse cardiac phenotype of the Hippo signaling pathway (Table 1).

**Table 1:** Mouse cardiac phenotypes in the activated or inactivated state of Hippo signaling

Genes	Models	Promoters	Stages	Cardiac phenotypes	Ref.
Salv	CKO	Nkx2.5 Cre	embryo	Elevated cardiomyocyte proliferation and cardiomyocyte numbers, hypertrophied hearts, some with VSD, dilated myocardium, thickened ventricular walls, and enlarged walls.	[127]
Lats2 or Mst1/2	CKO	Nkx2.5 Cre	embryo	Dilated myocardium, thickened ventricular walls, and enlarged ventricular walls at E11.5.	[127]
YAP	CKO	Nkx2.5 Cre	embryo	Inhibited cardiomyocyte proliferation, cardiac dysplasia and lethality at E10.5.	[128]
YAP	OE	β-MHC	embryo	Increased cardiomyocyte proliferation and cardiomyocyte number as well as heart size, dilated trabecular layer and adverse thickening of the myocardium.	[128]
YAP1	CKO; OE	Tnnt2 Cre	embryo/ neonate	In YAP1 CKO mice, inhibited cardiomyocyte proliferation and lethal myocardial dysplasia; in YAP1 OE mice, increased cardiomyocyte proliferation.	[126]
YAP	CKO	α-MHC Cre	adult/ neonate	Fibrosis, impaired cardiac function and myocardial regeneration.	[129]
YAP1	CKO	α-MHC Cre	adult	In homozygous knockout mice, increased cardiomyocyte apoptosis, fibrosis and premature death; in heterozygous knockout mice, decreased cardiomyocyte proliferation, increased cardiomyocyte apoptosis, impaired cardiac function and myocardial fibrosis after MI.	[130]
Lats1/2 or Salv	CKO	Myh6-Cre/Esr1	P8/adult	In Lats1/2 or Salv CKO adult mice, increased cardiomyocyte proliferation. in Salv CKO mice, increased cardiomyocyte renewal, reduced fibrotic area and improved cardiac function after AR in P8 mice; in Salv CKO mice, reduced infarct area and improved cardiac function after MI in P8 and adult mice.	[131]
YAP	OE	Myh6-Cre	adult	Improved cardiomyocyte proliferation and survival, reduced scar size and repaired heart function after MI.	[134]
Salv	CKO	Mhy6-Cre^ert^	P8/adult	Increased cardiomyocyte renewal after MI.	[132]
Salv	CKO	α-MHC-mcm	adult	Increased peri-scar revascularization, reduced infarct size, and improvement in pumping function after iHF.	[135]
Salv	CKO	Myh6-Cre^ert^	P8	Increased cardiomyocyte proliferation, improved fibrosis and cardiac function after AR.	[133]

*CKO* cardiac-specific knockout, *iHF* ischemic heart failure, *MI* myocardial infarction, *OE* overexpression, *P8* postnatal day 8, *VSD* ventricular septal defects.

There are many discussions about the molecular mechanisms of the Hippo/YAP signaling pathway. It was shown that YAP-TEAD interactions were critical in Hippo signaling-mediated organ development [126]. Lin et al. found that in neonatal mouse hearts, inhibition of acetylation of VGLL4 (as a TEAD1-binding protein) at the lysine 225 site promoted TEAD1 degradation thereby blocking the pro-proliferative effects of YAP-TEAD1 on cardiomyocytes [136]. In addition, the researchers revealed the expression of Park2 (Parkinson disease protein 2), a target gene of YAP, increased in Salv deleted adult hearts, suggesting the impact of mitochondrial quality control on cardiac repair [135]. Morikawa et al. reported that YAP was involved in the regulation of genes that linked proteins of the actin cytoskeleton to the extracellular matrix, including components of the DGC (dystrophin-glycoprotein complex). Its deficiency leads to severe muscular dystrophy. Besides, cardiomyocyte proliferation was not affected in Mdx (a model of muscular dystrophy) mice, but cytoskeletal remodeling was abnormal and thus cardiac regeneration was inhibited [132]. It demonstrates an important role for DGC in cardiac regeneration. Interestingly, the researchers discovered that DAG1 (a DGC component) could inhibit nuclear YAP content by binding to YAP, which was enhanced by activation of Hippo signaling, suggesting that the two could synergistically affect YAP. In addition, knockout Salv in the heart of Mdx mice exhibited a marked repair phenotype, cardiomyocyte overproliferation, and discordant tissue growth [133]. It indicates that DGC is critical for maintaining normal tissue morphology during the regeneration process after injury. In addition, YAP could activate PI3K/AKT signaling by promoting the expression of its target gene Pik3cb (a catalytic subunit of PI3K) to induce cardiomyocyte proliferation [137]. YAP could also activate the IGF (insulin-like growth factor) signaling of cardiomyocytes and inactivate GSK-3β, thereby increasing the level of β-catenin [128]. Additionally, the increased expression of Wnt target genes was observed in the Salv CKO heart, accompanied by a strong enhancement of nuclear β-catenin signaling, and the absence of β-catenin could rescue the overgrowth of the heart caused by Hippo signaling [127]. Taken together, Hippo/YAP signaling is closely related to Wnt/β-catenin and PI3K/AKT signaling pathways in regulating cardiomyocyte proliferation and controlling organ size.

### Wnt/β-catenin signaling

Activation of the classical Wnt signaling pathway is mainly dominated by downstream effectors β-catenin, including Wnt ligands, Wnt receptors (Frizzled, LRP5/LRP6), destruction complexes (Axin, APC, GSK-3β, and CK1), and intranuclear β-catenin (detailed in Fig. 3). In brief, palmitoylation of Porcupine transfers Wnt to the plasma membrane and Wntless secretes it. Then, Wnt binds to membrane receptors to inhibit the degradation of β-catenin mediated by the destruction complex in the cytoplasm, resulting in the increase of β-catenin in the nucleus and promotes its binding to TCF/LEF transcription factors to regulate the expression of target genes [138]. Several studies have shown that Wnt signaling can regulate cell proliferation and cardiac regeneration, but the conclusions seem to be inconsistent.

The Wnt/β-catenin signaling pathway is closely related to the formation of the second heart field of the embryo, and intracytoplasmic β-catenin levels have a critical impact on myocardial proliferation during the early stages of cardiogenesis [139, 140]. In zebrafish hearts, Wnt/β-catenin signaling was activated during regeneration. Enhanced Wnt/β-catenin signaling mediated by deletion of axin1 or overexpression wnt8 (a classical Wnt member) promoted fin regeneration, and conversely, suppressed Wnt/β-catenin signaling mediated by overexpression of wnt5b (a non-classical Wnt member) inhibited fin regeneration [139]. Due to the pivotal role of GSK-3β in this pathway, the researchers found that *in vitro* administration of CHIR-99021 (a GSK-3β inhibitor) strongly increased nuclear β-catenin levels and promoted significant proliferation not only in human atrial myocytes but also in adult mouse cardiomyocytes [140, 141].. *In vivo*, myocardial-specific knockout of GSK-3β facilitated the proliferation of cardiomyocytes in adult mice after MI and pressure overload and inhibited left ventricular dilation to protect heart function after MI [142]. Similarly, BIO, another GSK-3β inhibitor, was found to increase positive cell cycle regulators and decrease expression of the CDK inhibitor p27 to induce proliferation of neonatal and adult rat cardiomyocytes *in vitro* [143]. *In vivo*, BIO could stimulate myocardial cell proliferation in zebrafish after HF and relieve myocardial fibrosis in rats after MI [144]. It was recently shown that Wnt/β-catenin signaling enhanced cardiomyocyte proliferation in neonatal mice but not in adult mice and that these two differential effects were mainly due to different core transcriptional networks driven by β-catenin [25]. Xin et al. found that Wnt was closely linked to IGF signaling in addition to Hippo signaling, as IGF signaling activation led to the inactivation of GSK-3β and the increase of β-catenin content [128]. Studies have shown that IGF signal is also essential for cardiomyocyte proliferation and cardiac repair [145]. Yu et al. revealed IGF-1 reversed the H9C2 cell cycle arrest caused by high glucose and that both PI3K inhibitors and β-catenin inhibitors could inhibit this effect [146]. It indicates that IGF-1 at least partly plays its role through PI3K/β-catenin. Moreover, Wang et al. identified a novel regulator of myocardial cell proliferation, an IGF signaling pathway-related gene, IGF2BP3, which was enriched in the regenerative heart of myocardial infarction. Overexpression of IGF2BP3 has been confirmed to activate cardiomyocyte division and may be a potential target for future cardiac regeneration therapy [147]. Although the specific mechanism by which IGF2BP3 promotes heart regeneration has not been reported, we speculate that it must be related to the activation of the IGF pathway.

However, activation of Wnt canonical signaling inhibits cardiomyocyte proliferation and heart regeneration in some models. Zhao et al. found that after heart injury, the activation of Wnt signaling retarded heart regeneration in zebrafish, while the expression of Notum1b and Wif1 (a Wnt antagonist) promoted the renewal of regenerated heart cardiomyocytes [103]. In addition, either conditional deletion of β-catenin or administration of ICG-001 (a small molecule Wnt/β-catenin signaling inhibitor) improved cardiac function after MI; the former also could inhibit abnormal ventricular remodeling and the latter could drive tissue repair after injury [148, 149]. Similarly, the small molecule CDMG (a Wnt inhibitor) could affect cardiac regenerative capacity by promoting cardiomyocyte proliferation and infarct size reduction after injury in zebrafish and mammals [150]. Another small molecule inhibitor of Wnt signaling, Pyrvinium, increased the number of Ki67-positive cardiomyocytes in the border zone and remote zone of MI and inhibited adverse ventricular remodeling [151]. sFRP2, as a member of soluble Frizzled-related protein, is an extracellular inhibitor of Wnt. Alfaro et al. found that injection of MSCs overexpressing sFRP2 into the peri-infarct myocardium significantly reduced the area of fibrosis and enhanced cardiac function after MI [152]. Besides, Bastakoty et al. showed the inactivation of the Wnt pathway caused by GNF-6231 (a Porcupine inhibitor) rescued poor ventricular remodeling and improved cardiac function after AMI and that the Wnt inhibitor C-113 (CK1α activator) did not affect BrdU incorporation in HL-1 cardiomyocytes [153]. Yang et al. demonstrated that CGX1321 (another Porcupine inhibitor) had similar protective effects on the heart such as reducing scar area and improving cardiac function. But unlike C-113, CGX1321 could contribute to an increase in the number of EdU-positive cardiomyocytes in the infarct margins and an increase in the number of Ki67/pH3-positive cardiomyocytes *in vitro* [154]. It suggests that CGX1321 can target cardiomyocyte proliferation to stimulate cardiac regeneration.

It seems that we can find controversial effects of the Wnt/β-catenin signaling pathway on cardiac regeneration. There is no doubt that Wnt/β-catenin signaling is necessary during cardiac development. However, the findings after birth are not uniform. After comparing several studies, we do not obtain a clear rule, but this does not mean that the findings are wrong; on the contrary, it reminds us of the complexity of the heart regeneration process. We believe that it may be due to the following reasons: 1. There is crosstalk in each signal pathway, and the same kinase may participate in several pathways, so heart regeneration after an injury cannot be completely attributed to a single role of a signal pathway. 2. The signal pathway may be related to apoptosis, fibrosis, inflammation, angiogenesis, migration, and adhesion regulation. It is the result of multiple effects. Heart regeneration is not entirely the regeneration of cardiomyocytes. 3. Neither the administration of small molecules nor the deletion or overexpression of Wnt pathway components can guarantee that only the Wnt classical signaling pathway is acted upon. Furthermore, whether the systemic or myocardial local action has an impact on the final effect. A study showed that myocardial-specific overexpression of Dvl-1 resulted in the activation of both classical and nonclassical Wnt signaling and exhibited marked cardiac hypertrophy with impaired cardiac function and even premature death, and found that Dvl-1 knockdown abolished the β-adrenergic-induced hypertrophic response [155]. It implicates activation of Wnt signaling as a key player in the generation of cardiac hypertrophy. Thus, whether the balance of Wnt signaling after an injury is tilted toward hypertrophy or regeneration is the result of multiple effects, and identifying the key factors or mechanisms that influence this balance is what we urgently need to do.

## Soluble factors

In addition to the insoluble extracellular matrix, soluble factors also play an important role in cardiac repair and regeneration. Soluble factors mainly refer to cytokines, including growth factor (GF), interleukin (IL), tumor necrosis factor (TNF), colony-stimulating factor (CSF), chemokines, etc. They are a class of proteins that transduce intercellular signals by binding to corresponding receptors, thus exerting biological effects such as regulation of cellular immunity and growth. Next, we summarize the soluble factors that have been involved in the cardiac regeneration process in recent years.

### Growth factors

A study showed that pharmacological inhibition and mechanical damage to nerves led to impaired regeneration in zebrafish and mouse hearts and that neuregulin 1 (NRG1) and nerve growth factor (NGF) recombinant proteins rescued this model [156]. It implies that NRG1 and NGF may have key roles in the regeneration process. Lam et al. reported that NGF was sufficient to rescue aristolochic acid-induced lethal HF in zebrafish by targeting cardiomyocyte proliferation and reducing apoptosis to enhance regenerative capacity [157]. Furthermore, Gemberling et al. observed NRG1 was significantly present in perivascular cells of the post-injury heart in adult zebrafish, NRG1 overexpression promoted the proliferation of myocardial cells in the injured heart and the significant myocardial hyperplasia and even hypertrophy in the non-injured heart, so the researchers defined it as a mitogen that induced efficient myocardial regeneration [90]. In a recent study, Shoffner et al. validated the role of NRG1 as a mitogen in the zebrafish heart [158]. This effect has also been demonstrated in mammals. A study showed that intracardiac injection of NRG1 microparticles improved post-myocardial infarction angiogenesis and cardiac function in rats [159]. Another study showed that recombinant NRG1 not only promoted the proliferation of cardiomyocytes in adult mice but also had a better effect on repairing heart function after injury when administered in neonates than in adults. In addition, the administration of recombinant NRG1 could also stimulate the proliferation of cardiomyocytes in children with heart disease within 6 months of birth [160].

In the zebrafish heart, the stress response impeded cardiac regeneration and downregulated IGF (insulin-like growth factor) signaling genes, suggesting that impaired stress-induced cardiac regeneration was associated with IGF signaling [161]. The researchers found IGF-2 played an important role not only in mouse heart development but also in zebrafish heart regeneration and that inhibition of IGF-1R hindered cardiomyocyte proliferation during both developmental and regenerative stages [145]. Shen et al. recently reported that IGF-2 abolished the intrinsic regenerative effects in P1-day mice and revealed the important identity of IGF-2 as a mitogen in neonatal mice [162]. In addition, IGF-1 and HGF (hepatocyte growth factor) combination in mouse MI hearts greatly increased the survival of Sca-1+/CD31- transplanted cells and significantly promoted cardiac regeneration [163].

There are still the following growth factors related to heart regeneration. Lien et al. reported that PDGF (platelet-derived growth factor) signaling played a crucial role in zebrafish heart regeneration; specifically, the expression of PDGF-A and PDGF-B increased during regeneration, and inhibition of PDGF receptors attenuated the DNA synthesis of myocytes after injury [164]. Moreover, MYDGF (myeloid-derived growth factor) up-regulated in the myocardium of neonatal mice after injury, and overexpression of MYDGF stimulated cardiomyocyte proliferation and myocardial regeneration in neonatal and adult mice by targeting the c-Myc/FoxM1 pathway [165]. FGF (fibroblast growth factor) signaling was also involved in regulating cardiac regeneration, and its receptor FGFR-1 interacted with Fn14 (fibroblast growth factor-inducible molecule) to induce the restart of cell cycle progression [166]. A study showed that intracardiac injection of FGF1 microparticles promoted angiogenesis and recovery of cardiac function after myocardial infarction in rats [159]. Mechanistically, the regenerative role of FGF signaling was associated with the activation of AKT [166, 167]. However, in practical applications, growth factors will degrade in the tissues and affect the effectiveness of the therapeutic effect. Researchers have found that the use of PLGA and PEG-PLGA microparticles can solve this problem, which is beneficial to the clinical application of growth factors [159].

### Other soluble factors

Although interleukins are mainly associated with inflammation, there are also some reports on the regulation of proliferation by interleukins. Overexpression of IL-6 (interleukin-6) affected the expression of genes related to cell cycle progression and thus stimulated cardiomyocyte proliferation and recovery of cardiac morphology and function after injury [168]. Additionally, a member of the IL-6 family, OSM, was closely associated with cardiac regeneration in neonatal mice, as evidenced by the fact that deletion of both OSM and its receptor gp130 significantly inhibited cardiomyocyte proliferation and myocardial regeneration [169]. Another key factor in the regeneration process is IL-13. Administration of IL-13 reversed the regeneration impairment caused by myocardial-specific knockout of GATA4 [52, 170]. Subsequently, it was shown that IL-13 affected cell proliferation, apoptosis, and regeneration in neonatal mouse hearts by targeting ERK1/2 and Akt signaling [171]. Besides, several studies have shown that IL-4 is involved in regulating the proliferation of neonatal murine cardiomyocytes [172]; IL-7 can greatly enhance the cardiac function of MSCs transplantation and improve the heart morphology compared with only transplanted MSCs [173]; IL-15 also significantly protects cardiac function in mice after MI by reducing infarct size and promoting angiogenesis [174]. The above results demonstrate that the immune response affects cardiac regeneration.

TWEAK, a member of the TNF (tumor necrosis factor) family, promoted the expression of pH3, Aurora B, Ki67-positive cardiomyocytes, and cell cycle-related genes in neonatal rats by activating ERK/PI3K and inhibiting GSK-3β [175]. In addition, TWEAK stimulation with the expression of its receptor FN14 also had a proliferative effect on adult cardiomyocyte proliferation [175]. Although this effect did not appear to be related to cardiomyocyte proliferation, G-CSF (granulocyte colony-stimulating factor) did exert cardioprotective effects directly on the myocardium by reducing inflammatory factors and cardiomyocyte/endothelial cell apoptosis, and by alleviating adverse cardiac remodeling and myocardial dysfunction post-MI [176, 177]. Itou et al. found cardiac injury in zebrafish activated the expression of the chemokine ligand Cxcl12a and its receptor Cxcr4b and that inhibition of Cxcr4 affected cell migration and loss of Cxcr4b function impeded cardiac regeneration in a manner that did not affect cardiomyocyte proliferation [178]. It suggests that migration is also essential for cardiac regeneration. Additionally, a study showed that recombinant Cxcl8 and Ccl2 rescued GM6001 (a matrix metalloproteinase inhibitor) induced regeneration impairment in zebrafish after cryoinjury [179]. Wang et al. unearthed data on cardiac regeneration after injury by comparing the changes of histones in the epigenome in the regenerative and non-regenerative hearts of mice, and they identified Ccl24 as a cardiac regeneration-related gene [147]. This implies that we can restart the regeneration process of the damaged heart by targeting the embryonic heart gene program in the regeneration window.

## MicroRNAs

MicroRNAs are single-stranded small molecule RNAs encoded by endogenous genes with a length of about 22-25 nucleotides, which have the function of regulating the expression of genes at translation level or post-transcriptional level. As an important non-coding RNA, microRNAs which play vital roles in cardiovascular diseases are a striking contributor to the proliferation of postnatal cardiomyocytes in mice.

The miR-17-92 cluster serves as a highly conserved gene cluster that encodes a variety of conserved miRNAs [180]. Researchers found that miR-17-92 was a critical regulator in inducing cardiomyocyte proliferation in embryonic, neonatal, and adult myocardial tissue. *In vivo,* miR-17-92 enhancement was beneficial to the proliferation of myocardial cells and heart repair following myocardial infarction in mice. *In vitro*, overexpression of miR-19a/19b significantly increased DNA synthesis, cytokinesis, and the expression of CDK1 mRNA, which was mediated by PTEN. Overexpression of PTEN could completely reverse the proliferation caused by miR-19a /19b [181, 182].

The expression of miR-34a was not easily detected at birth but was significantly up-regulated in postnatal day 7 hearts and adult hearts MI. In the early neonatal heart, overexpression of miR-34a inhibited the ability of cardiomyocyte proliferation and cardiac regeneration post-injury. In adult hearts, knockdown of miR-34a accelerated cell cycle activity and heart repair after myocardial infarction [183]. Additionally, repressing miR-34a could diminish myocardial fibrosis and cardiomyocyte apoptosis by regulating PNUTS, and could facilitate the recovery of cardiac contractility after acute myocardial infarction [184].

MiR-210 has a protective effect on ischemic injury, and multiple conserved target genes of miR-210 have correlated with DNA repair, cell cycle progression, and angiogenesis [185]. Arif revealed that miR-210 induced proliferation and a sharp reduction of cell death by directly targeting APC (adenomatous polyposis coli) in adult rat cardiomyocytes. Overexpression of miR-210 promoted the division of cardiomyocytes following ischemic injury or MI in mice. Moreover, miR-210 overexpression also recovered the heart function, reduced cell apoptosis, and increased neovascularization in the mature myocardium after myocardial infarction [186].

A study showed that knockdown of miR-128 inhibited cardiomyocyte apoptosis after ischemia-reperfusion injury in rabbits [187]. Huang et al. firstly confirmed that cardiac-specific overexpression of miR-128 undermined myocardial homeostasis and myocardial cell proliferation and attenuated cardiac regeneration and heart function in postnatal hearts. Conversely, the silencing of miR-128 stimulated cardiomyocyte duplication in postnatal mice and heart regeneration in adult mice, inhibited the expression of the fibrosis gene, and relieved heart failure post-MI. Mechanistically, miR-128 changed the expression of chromatin modifier SUZ12 and consequently affected the expression of genes related to the cell cycle [188].

MiR-294, which belongs to embryonic stem cell cycle (ESCC) miRNAs, was a cell cycle regulator that was expressed in the embryo and early development of the heart and had a strong ability to promote cardiac myocyte proliferate [189]. *In vitro*, overexpression of miR-294 triggered cell cycle reentry by targeting the Wee1/CDK1-CyclinB1 axis, and the expression of cell cycle proliferation markers, oxidative phosphorylation, and glycolysis was dramatically enhanced. *In vivo*, miR-294 activation for two consecutive post-myocardial ischemia decreased the area of myocardial scarring and enhanced cardiac function. Simultaneously, the number of cardiomyocytes in miR-294 overexpression hearts was increased, accompanied by enhanced expression of EdU, pH3, and Aurora B positive myocytes. They also revealed that the adult cardiomyocytes activated by miR-294 were neither in an adult state nor an embryonic state but in an intermediate state capable of producing new cells [190]. Therefore, the enhancement of cardiac repair in response to myocardial infarction may be due to the increased proliferation of cardiomyocytes in the intermediate state.

## Discussion

**Fig. 4:**
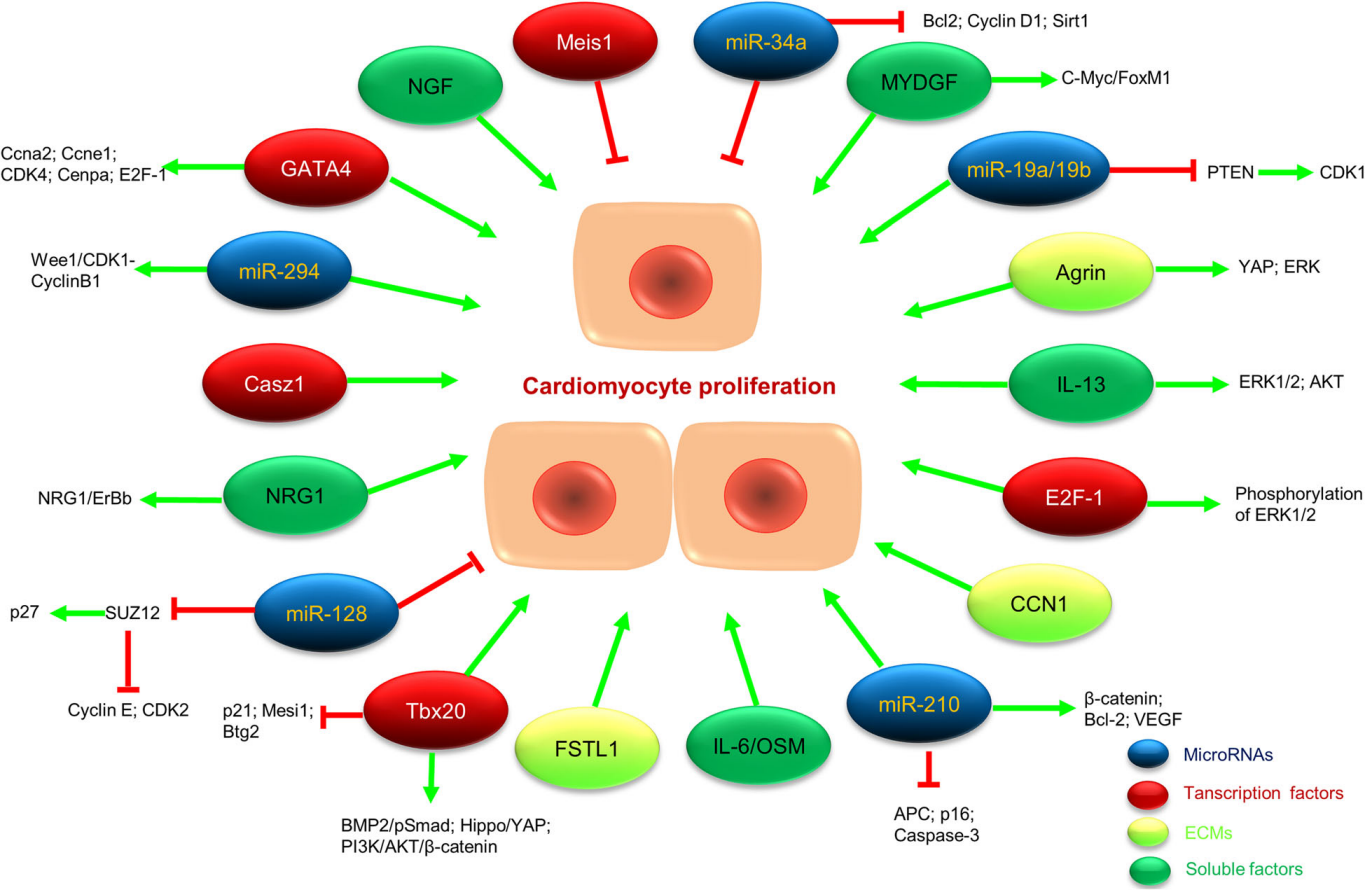
Major factors that target cardiomyocyte proliferation and their possible molecular pathways. Green arrows indicate that cardiomyocyte proliferation or gene expression is promoted, and red solid lines indicate that cardiomyocyte proliferation or gene expression is inhibited. Bcl-2: B-cell lymphoma-2; Btg2: B-cell translocation gene 2; BMP2: bone morphogenetic protein-2; Ccna2: cyclin A2; Ccne1: cyclin E1; Cenpa: centromere protein A; CDK1/2/4: cyclin-dependent kinase 1/2/4; C-Myc: cancer MYC; E2F-1: FoxM1: forkhead box protein M1; pSmad: phosphorylated mothers against DPP homolog; PTEN: phosphatase and tensin homologue deleted on chromosome 10; p27/ p21/ p16: cyclin-dependent kinase inhibitor; SUZ12: suppressor of Zeste 12 homolog; Sirt1: Sirtuin 1; VEGF: vascular endothelial growth factor; Wee1: G2 checkpoint kinase

A brief review of the literature exemplifies the multi-level regenerative regulation of proteins, non-coding RNAs and signaling pathways, etc. Taken together we believe that cardiac regenerative repair is governed by regenerationomics which is a dynamic regulatory network including the major factors modulating the proliferation of adult mammalian cardiomyocytes (Fig. 4), which will provide new ideas for regenerative medicine research.

As we know, not all regeneration is mediated by cardiomyocyte proliferation. Although targeting cardiomyocyte proliferation is a hot topic of research in the field of regeneration, it was found that for cardiac regeneration, cell migration and mechanical changes in the cytoskeleton are also important [94, 132, 178]. Additionally, paracrine factors have a significant cofactor effect on cardiac regeneration after injury [163, 173]. This reminds us that cardiac regeneration is a complex process that cannot be attributed to a single factor and a single cell type. Therefore, two urgent puzzles need to be solved as follows. One is which of these regenerative regulators has the strongest effect and which factor dominates when acting simultaneously? The other question is whether the combined application of regeneration regulators will enhance regenerative capacity or create other negative crosstalk? What combination will maximize the effect? Finally, it is suggested that studies focus more on the combined application of multiple myocardial regeneration regulators compared to the discovery of new single regulatory factors.

Furthermore, the dose conversion of regenerative drugs is also one of the challenges we face. MiR-199a could promote the regeneration of mouse cardiomyocytes [191, 192], reduce infarct size by 50%, and exert an almost complete recovery of the ejection fraction when applied to pigs. Nevertheless, seven animals suddenly expired 7 to 8 weeks after infarction [193]. Therefore, the issue how to apply RNA delivery therapy to large animals rather than mice needs to be solved urgently. The therapeutic dose conversion of microRNAs between mice and large animals and even humans remains to be studied. In short, the findings suggest that there are still many unknown dilemmas in terms of cardiac regeneration waiting for us to explore.

The value of scientific research is to benefit human beings, so virus-mediated gene therapy is an inevitable part. In recent years, gene therapy mediated by expensive adeno-associated virus (AAV) has crucial effects and was shown not to cause any human disease compared to retroviruses and adenoviruses [194]. However, continuous research showed AAV could cause cancer by inserting an exogenous therapeutic gene fragment near the gene that controls growth [195]. Even though gene therapy comes with tremendous risks, if we can overcome this problem or discover new safe and non-toxic gene therapies, it will be a key step towards clinical treatment.

Therefore, there is still a long way to go before these regenerative factors can be applied in the clinic. Before that, we must deepen our understanding and clarify the molecular mechanisms of cardiac regeneration to truly identify effective methods for regenerating the adult mammalian heart. Despite there are still mysteries in the research, the latest advances in endogenous-mediated cardiac regeneration provide the basis for introducing these factors into clinical treatment in the future and may bring a silver lining to the treatment of cardiovascular diseases in humans.

## Data Availability

Not applicable.

## Abbreviations

AR: Apical resection
APC: Adenomatous polyposis coli
BIO: 6-bromoindirubin-39-oxime
BrdU: 5-bromo-2′-deoxyuridine
Bcl-2: B-cell lymphoma-2
Btg2: B-cell translocation gene 2
BMP2: Bone morphogenetic protein-2
CSL: CBF1/Su(H)/Lag-1
CCN1: Cysteine-rich 61
Ccna2: Cyclin A2
Ccne1: Cyclin E1
Cenpa: Centromere protein A
CDK1/2/4: Cyclin dependent kinase 1/2/4
C-Myc: Cancer MYC
CK1: Casein kinase-1
CDMG: Cardiomogen
Cxcl12a: Chemokine (C-X-C motif) ligand 12a
Cxcl8: Chemokine (C-X-C motif) ligand 8
Cxcr4b: Chemokine (C-X-C motif) receptor 4b
Ccl2: C-C motif chemokine 2
Ccl24: C-C motif chemokine 24
DAG1: Dystroglycan 1
Dvl: Dishevelled
ERK: Extracellular receptor kinase
ERK1/2: Extracellular signal-regulated kinases 1 and 2
EdU: 5-ethynyl-2'-deoxyuridine
ECRAR: Endogenous cardiac regeneration-associated regulator
E2F-1/2: E2F transcription factor 1/2
FoxM1: Forkhead box protein M1
FSTL-1: Follistatin-like 1
FN14: Fibroblast growth factor-inducible molecule 14 receptor
GSK3: Glycogen synthase kinase-3
G-CSF: Granulocyte colony-stimulating factor
Hoxb13: Homeobox b13
HGF: Hepatocyte growth factor
IGF: Insulin-like growth factor
IGF2BP3: Insulin-like growth factor 2 messenger RNA-binding protein 3
iHF: Ischemic heart failure
IGF: Insulin-like growth factor
LRP5/6: Low-density lipoprotein receptor-related protein 5/6
LATS: Large tumour suppressor homologue
MI: Myocardial infarction
MYDGF: Myeloid-derived growth factor
Meis1: Meis homeobox 1
MSCs: Mesenchymal stem cells
MOB1: MOB kinase activator 1
MST: Mammalian STE20-like protein kinase
NRG-1: Neuregulin-1
NGF: Nerve growth factor
Notum1b: Palmitoleoyl-protein carboxylesterase 1b
OSM: Oncostatin M
pSmad: Phosphorylated mothers against DPP homolog
PTEN: Phosphatase and tensin homologue deleted on chromosome 10
Park 2: Parkinson disease protein 2
pH3: Phosphorylation of histone 3
PI3K: Phosphoinositide 3-kinase
PLGA: Poly (lactic-co-glycolic acid)
PEG-PLGA: Poly (lactic-co-glycolic acid) and polyethylene glycol
PDGF: Platelet-derived growth factor
p27/ p21/ p16: Cyclin dependent kinase inhibitor
sFRP: Soluble Frizzled-related protein
SAV1: Protein salvador homologue 1
SUZ12: Suppressor of Zeste 12 homolog
Sirt1: Sirtuin 1
TAZ: Transcriptional coactivator with PDX-binding motif
TCF/LEF: T-cell factor/lymphoid enhancer-binding factor
TEADs: TEA domain transcription factor family members
TWEAK: TNF-related weak inducer of apoptosis
TNF: Tumor necrosis factor
VEGF: Vascular endothelial growth factor
Wif1: Wnt inhibitory factor 1
WNT: Wingless/int-1 protein
Wee1: G2 checkpoint kinase
YAP: Yes-associated protein
